# Bis(4-amino­benzoic acid-κ*N*)dichloridozinc(II)

**DOI:** 10.1107/S1600536810047902

**Published:** 2010-11-24

**Authors:** Melanie Rademeyer, Gerhard E. Overbeek, David C. Liles

**Affiliations:** a Department of Chemistry, University of Pretoria, Pretoria 0002, South Africa

## Abstract

Mol­ecules of the title compound [ZnCl_2_(C_7_H_7_NO_2_)_2_], are located on a twofold rotation axis. Two 4-amino­benzoic acid moieties, and two chloride ligands are coordinated to a Zn atom in a tetra­hedral fashion, forming an isolated mol­ecule. Neighbouring mol­ecules are linked through hydrogen-bonded carboxyl groups, as well as N—H⋯Cl hydrogen-bonding inter­actions between amine groups and the chloride ligands of neighbouring mol­ecules, forming a three-dimensional network.

## Related literature

For a related structure, see: Wang *et al.* (2002 ▶). For hydrogen-bond motifs, see: Bernstein *et al.* (1995 ▶).
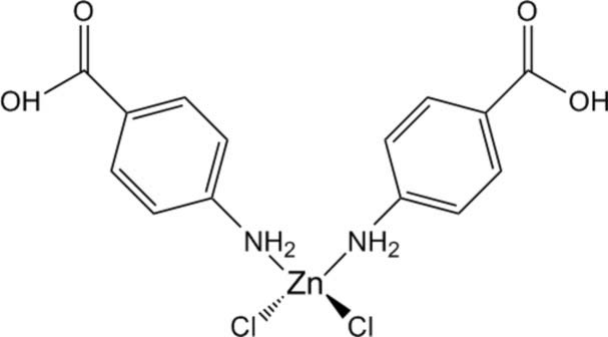


## Experimental

### 

#### Crystal data

[ZnCl_2_(C_7_H_7_NO_2_)_2_]
*M* *_r_* = 410.56
Monoclinic, 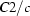

*a* = 30.646 (2) Å
*b* = 4.7248 (3) Å
*c* = 11.6157 (8) Å
β = 97.089 (1)°
*V* = 1669.05 (19) Å^3^
*Z* = 4
Mo *K*α radiation
μ = 1.81 mm^−1^
*T* = 293 K
0.42 × 0.09 × 0.07 mm

#### Data collection

Bruker (Siemens) P4 diffractometer
Absorption correction: multi-scan (*SADABS*; Bruker, 2001 ▶) *T*_min_ = 0.769, *T*_max_ = 0.881
4246 measured reflections
1571 independent reflections
1467 reflections with *I* > 2σ(*I*)
*R*_int_ = 0.026

#### Refinement

*R*[*F* ^2^ > 2σ(*F* ^2^)] = 0.029
*wR*(*F* ^2^) = 0.077
*S* = 1.15
1571 reflections
105 parameters
H-atom parameters constrained
Δρ_max_ = 0.33 e Å^−3^
Δρ_min_ = −0.23 e Å^−3^

### 

Data collection: *SMART* (Bruker, 2001 ▶); cell refinement: *SAINT* (Bruker, 2001 ▶); data reduction: *SAINT*; program(s) used to solve structure: *SHELXS97* (Sheldrick, 2008 ▶); program(s) used to refine structure: *SHELXL97* (Sheldrick, 2008 ▶); molecular graphics: *ORTEP-3 for Windows* (Farrugia, 1997 ▶) and *Mercury* (Bruno *et al.*, 2002 ▶); software used to prepare material for publication: *PLATON* (Spek, 2009 ▶) and *WinGX* (Farrugia, 1999 ▶).

## Supplementary Material

Crystal structure: contains datablocks global, I. DOI: 10.1107/S1600536810047902/bt5408sup1.cif

Structure factors: contains datablocks I. DOI: 10.1107/S1600536810047902/bt5408Isup2.hkl

Additional supplementary materials:  crystallographic information; 3D view; checkCIF report

## Figures and Tables

**Table 1:** Hydrogen-bond geometry (Å, °)

*D*—H⋯*A*	*D*—H	H⋯*A*	*D*⋯*A*	*D*—H⋯*A*
O2—H1⋯O1^i^	0.80	1.82	2.609 (3)	170
N1—H1*A*⋯Cl1^ii^	0.90	2.64	3.5028 (17)	162
N1—H1*B*⋯Cl1^iii^	0.90	2.60	3.3978 (17)	148

Symmetry codes: (i) 
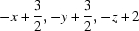
; (ii) 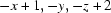; (iii) 
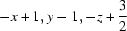
.

**Fig. 1.**
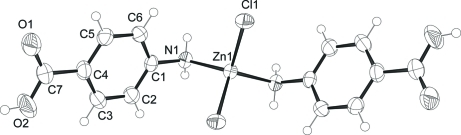
The asymmetric unit of I showing the atomic numbering scheme. Displacement ellipsoids are shown at the 50% probability level and H atoms are shown as small spheres of arbitrary radii.

**Fig. 2.**
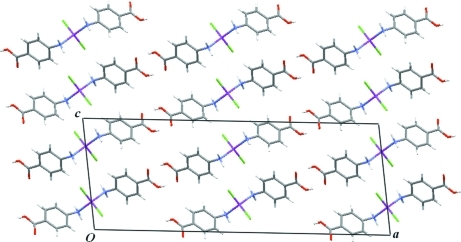
Packing diagram of I viewed down the b-axis.

**Fig. 3.**
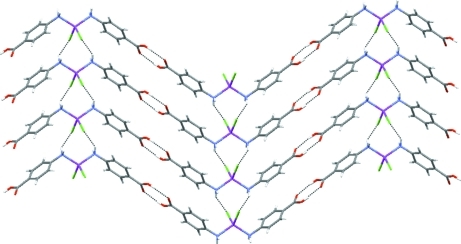
O—H···O and N—H···Cl hydrogen bonding interactions link molecules to form a two-dimensional hydrogen bonded sheet.

## supplementary crystallographic information

### Comment

The crystal structure of dichloro-bis(4-aminobenzoic acid-N)-zinc(ii), I, was determined as part of an ongoing study of the coordination compounds formed between organic amines and metal halides. The related crystal structure of diiodo-bis(4-aminobenzoic acid-N)-cadmium(ii) has been reported (Wang *et al.*, 2002), but the crystal structures are not isostructural.

The asymmetric unit of I consists of one 4-aminobenzoic acid moiety coordinated to a ZnCl unit through the nitrogen atom, as shown in Fig. 1, with the Zn atom lying on a twofold rotation axis. The second half of the molecule is generated by the symmetry operator (*-x, y, 1/2 - z*), and the unit cell contains four dichloro-bis(4-aminobenzoic acid-N)-zinc(II) molecules.

The Zn atom is coordinated to two 4-aminobenzamide ligands, through the nitrogen atom, and to two chloro ligands, and displays a slightly distorted tetrahedral coordination geometry with the N—Zn—N angle equal to 114.99 (9)°, which is slightly larger than the ideal tetrahedral angle of 109.5° to reduce steric hinderance between the two bulky 4-aminobenzoic acid ligands. The N—Zn—Cl angles adopt values of 107.10 (5)° and 109.27 (5)°, while the Cl—Zn—Cl angle has a value of 109.00 (3)°. The 4-aminobenzoic acid ligands show a *cis* orientation relative to the Zn atom, and in each ligand the aromatic plane forms an angle of 2.7 (0.1)° relative to the carboxylic acid group plane, rendering the ligand non-planar.

The layered packing of the molecules parallel to the *bc*-plane is illustrated in Fig. 2. The aromatic rings pack in two layers, while the Cl—Zn—Cl moieties form a layer. Hydrogen bonding interactions between the carboxylic acid groups of neighbouring layers result in the formation of carboxylic acid dimers of graph set notation *R*_2_^2^(8) (Bernstein *et al.*, 1995) on both sides of the molecule, forming a zigzag, one-dimensional hydrogen bonded ribbon as shown in Fig. 3. Neighbouring one-dimensional ribbons are connected *via* N1—H1B···Cl1 (symmetry operator: *-x + 1, y - 1, -z + 3/2*) hydrogen bonds to form the two-dimensional hydrogen bonded sheet illustrated in Fig. 3, with the intra-ribbon interactions described by the graph set notation *R*_2_^2^(8). Additional N1—H1B···Cl1 (symmetry operator: *-x + 1, -y, -z + 2*) hydrogen bonds link neighbouring sheets to give a three-dimensional hydrogen bonded structure, with intra-sheet hydrogen bonds described by the graph set notation D_1_^1^. Hydrogen bonding parameters are listed in Table 1.

### Experimental

Dichloro-bis(4-aminobenzoic acid-N)-zinc(ii) was prepared by dissolving 4.34 g Zn(NO_3_)_2_.6H_2_O (14.6 mmol, Sigma-Aldrich, 98%) and 1.00 g 4-aminobenzoic acid (7.29 mmol, Aldrich Chemistry, 99%) in a mixture of 50 ml distilled water and 50 ml e thanol (Merck, 99.5%). Dissolution was achieved by heating the solution in a beaker to approximately 60°C. Approximately 30 ml of the solution was transferred to a polytop vial, and one drop of HCl (Promark Chemicals, 32%) was added to the solution. Slow evaporation of the solvent mixture at room temperature gave yellow crystals of the title compound.

### Refinement

All H atoms, except the carboxylic acid group hydrogen atom, were refined using a riding model, with C—H distances of 0.93 Å and N—H distances of 0.90 Å, and *U*_iso_(H) = 1.2*U*_eq_(C) or 1.2*U*_eq_(N). The carboxylic acid hydrogen atom was placed as observed on the difference Fourier map, and not further refined, with *U_iso_*(H)=1.2*U_eq_*(O).

### Crystal data

**Table:**

[ZnCl_2_(C_7_H_7_NO_2_)_2_]	*F*(000) = 832
*M**_r_* = 410.56	*D*_x_ = 1.634 Mg m^−^^3^
Monoclinic, *C*2/*c*	Mo *K*α radiation, λ = 0.71073 Å
Hall symbol: -C 2yc	Cell parameters from 3472 reflections
*a* = 30.646 (2) Å	θ = 2.7–26.3°
*b* = 4.7248 (3) Å	µ = 1.81 mm^−^^1^
*c* = 11.6157 (8) Å	*T* = 293 K
β = 97.089 (1)°	Needle, yellow
*V* = 1669.05 (19) Å^3^	0.42 × 0.09 × 0.07 mm
*Z* = 4	

### Data collection

**Table:**

Bruker P4 diffractometer	1571 independent reflections
Radiation source: fine-focus sealed tube	1467 reflections with *I* > 2σ(*I*)
graphite	*R*_int_ = 0.026
Detector resolution: 8.3 pixels mm^-1^	θ_max_ = 26.5°, θ_min_ = 2.7°
φ and ω scans	*h* = −37→31
Absorption correction: multi-scan (*SADABS*; Bruker, 2001)	*k* = −2→5
*T*_min_ = 0.769, *T*_max_ = 0.881	*l* = −14→14
4246 measured reflections	

### Refinement

**Table:**

Refinement on *F*^2^	Primary atom site location: structure-invariant direct methods
Least-squares matrix: full	Secondary atom site location: difference Fourier map
*R*[*F*^2^ > 2σ(*F*^2^)] = 0.029	Hydrogen site location: inferred from neighbouring sites
*wR*(*F*^2^) = 0.077	H-atom parameters constrained
*S* = 1.15	*w* = 1/[σ^2^(*F*_o_^2^) + (0.0449*P*)^2^ + 0.8598*P*] where *P* = (*F*_o_^2^ + 2*F*_c_^2^)/3
1571 reflections	(Δ/σ)_max_ = 0.001
105 parameters	Δρ_max_ = 0.33 e Å^−^^3^
0 restraints	Δρ_min_ = −0.23 e Å^−^^3^

### Special details

**Table:**

Geometry. All e.s.d.'s (except the e.s.d. in the dihedral angle between two l.s. planes) are estimated using the full covariance matrix. The cell e.s.d.'s are taken into account individually in the estimation of e.s.d.'s in distances, angles and torsion angles; correlations between e.s.d.'s in cell parameters are only used when they are defined by crystal symmetry.
Refinement. Refinement of *F*^2^ against ALL reflections. The weighted *R*-factor *wR* and goodness of fit *S* are based on *F*^2^, conventional *R*-factors *R* are based on *F*, with *F* set to zero for negative *F*^2^. The threshold expression of *F*^2^ > σ(*F*^2^) is used only for calculating *R*-factors(gt) *etc*. and is not relevant to the choice of reflections for refinement. *R*-factors based on *F*^2^ are statistically about twice as large as those based on *F*, and *R*- factors based on ALL data will be even larger.

### Fractional atomic coordinates and isotropic or equivalent isotropic displacement parameters (Å^2^)

**Table:**

	*x*	*y*	*z*	*U*_iso_*/*U*_eq_	
O1	0.70214 (6)	0.6634 (5)	1.04917 (17)	0.0663 (5)	
O2	0.73024 (7)	0.5084 (5)	0.8932 (2)	0.0778 (7)	
H1	0.7524	0.5979	0.9059	0.093*	
Zn1	0.5000	0.05824 (6)	0.7500	0.02934 (14)	
Cl1	0.468360 (16)	0.33410 (10)	0.87344 (4)	0.03776 (16)	
N1	0.54928 (5)	−0.1758 (3)	0.84134 (14)	0.0318 (3)	
H1A	0.5398	−0.2431	0.9064	0.038*	
H1B	0.5557	−0.3247	0.7981	0.038*	
C1	0.58845 (6)	−0.0093 (4)	0.87199 (17)	0.0309 (4)	
C2	0.62214 (7)	−0.0150 (5)	0.8037 (2)	0.0439 (5)	
H2	0.6203	−0.1328	0.7391	0.053*	
C3	0.65871 (7)	0.1553 (6)	0.8315 (2)	0.0501 (6)	
H3	0.6814	0.1521	0.7853	0.060*	
C4	0.66159 (7)	0.3302 (5)	0.92789 (19)	0.0411 (5)	
C5	0.62723 (7)	0.3376 (5)	0.99492 (18)	0.0387 (5)	
H5	0.6288	0.4565	1.0591	0.046*	
C6	0.59051 (6)	0.1691 (4)	0.96695 (18)	0.0360 (4)	
H6	0.5674	0.1758	1.0117	0.043*	
C7	0.70070 (8)	0.5140 (6)	0.9599 (2)	0.0505 (6)	

### Atomic displacement parameters (Å^2^)

**Table:**

	*U*^11^	*U*^22^	*U*^33^	*U*^12^	*U*^13^	*U*^23^
O1	0.0514 (10)	0.0825 (14)	0.0654 (12)	−0.0282 (10)	0.0087 (9)	−0.0222 (11)
O2	0.0481 (11)	0.1041 (16)	0.0851 (15)	−0.0392 (11)	0.0233 (10)	−0.0285 (13)
Zn1	0.02820 (19)	0.0287 (2)	0.0304 (2)	0.000	0.00094 (13)	0.000
Cl1	0.0445 (3)	0.0359 (3)	0.0343 (3)	−0.0004 (2)	0.0108 (2)	−0.00444 (19)
N1	0.0320 (8)	0.0267 (8)	0.0351 (8)	−0.0014 (6)	−0.0016 (6)	0.0025 (6)
C1	0.0285 (9)	0.0281 (8)	0.0343 (10)	−0.0003 (7)	−0.0030 (8)	0.0048 (8)
C2	0.0378 (11)	0.0516 (12)	0.0424 (12)	−0.0025 (10)	0.0047 (9)	−0.0115 (10)
C3	0.0333 (11)	0.0677 (15)	0.0511 (14)	−0.0081 (11)	0.0125 (10)	−0.0099 (12)
C4	0.0310 (10)	0.0460 (12)	0.0450 (12)	−0.0077 (9)	−0.0001 (9)	0.0007 (10)
C5	0.0388 (11)	0.0401 (11)	0.0363 (11)	−0.0069 (9)	0.0011 (8)	−0.0034 (9)
C6	0.0338 (10)	0.0382 (11)	0.0365 (10)	−0.0041 (8)	0.0064 (8)	0.0000 (8)
C7	0.0367 (12)	0.0598 (14)	0.0548 (14)	−0.0138 (11)	0.0045 (10)	−0.0021 (12)

### Geometric parameters (Å, °)

**Table:**

O1—C7	1.250 (3)	C1—C6	1.383 (3)
O2—C7	1.262 (3)	C2—C3	1.385 (3)
O2—H1	0.80000	C2—H2	0.9300
Zn1—N1^i^	2.0577 (15)	C6—C5	1.384 (3)
Zn1—N1	2.0576 (15)	C6—H6	0.9300
Zn1—Cl1	2.2445 (5)	C5—C4	1.385 (3)
Zn1—Cl1^i^	2.2445 (5)	C5—H5	0.9300
N1—C1	1.443 (2)	C7—C4	1.490 (3)
N1—H1A	0.9000	C4—C3	1.386 (3)
N1—H1B	0.9000	C3—H3	0.9300
C1—C2	1.378 (3)		
			
C7—O2—H1	122.00	C1—C2—H2	120.1
N1^i^—Zn1—N1	114.98 (9)	C3—C2—H2	120.1
N1^i^—Zn1—Cl1	107.10 (5)	C1—C6—C5	119.57 (19)
N1—Zn1—Cl1	109.28 (5)	C1—C6—H6	120.2
N1^i^—Zn1—Cl1^i^	109.28 (5)	C5—C6—H6	120.2
N1—Zn1—Cl1^i^	107.10 (5)	C6—C5—C4	120.4 (2)
Cl1—Zn1—Cl1^i^	109.00 (3)	C6—C5—H5	119.8
C1—N1—Zn1	111.68 (11)	C4—C5—H5	119.8
C1—N1—H1A	109.3	O1—C7—O2	124.5 (2)
Zn1—N1—H1A	109.3	O1—C7—C4	118.8 (2)
C1—N1—H1B	109.3	O2—C7—C4	116.7 (2)
Zn1—N1—H1B	109.3	C5—C4—C3	119.5 (2)
H1A—N1—H1B	107.9	C5—C4—C7	119.3 (2)
C2—C1—C6	120.48 (19)	C3—C4—C7	121.2 (2)
C2—C1—N1	120.46 (19)	C2—C3—C4	120.3 (2)
C6—C1—N1	118.94 (18)	C2—C3—H3	119.9
C1—C2—C3	119.8 (2)	C4—C3—H3	119.9
			
N1^i^—Zn1—N1—C1	161.70 (15)	C6—C5—C4—C3	0.8 (3)
Cl1—Zn1—N1—C1	−77.86 (13)	C6—C5—C4—C7	179.9 (2)
Cl1^i^—Zn1—N1—C1	40.08 (14)	O1—C7—C4—C5	2.5 (4)
Zn1—N1—C1—C2	−95.8 (2)	O2—C7—C4—C5	−177.1 (3)
Zn1—N1—C1—C6	80.33 (19)	O1—C7—C4—C3	−178.4 (3)
C6—C1—C2—C3	1.2 (3)	O2—C7—C4—C3	2.0 (4)
N1—C1—C2—C3	177.2 (2)	C1—C2—C3—C4	0.2 (4)
C2—C1—C6—C5	−1.6 (3)	C5—C4—C3—C2	−1.2 (4)
N1—C1—C6—C5	−177.72 (18)	C7—C4—C3—C2	179.7 (2)
C1—C6—C5—C4	0.6 (3)		

Symmetry codes: (i) −*x*+1, *y*, −*z*+3/2.

### Hydrogen-bond geometry (Å, °)

**Table:**

*D*—H···*A*	*D*—H	H···*A*	*D*···*A*	*D*—H···*A*
O2—H1···O1^ii^	0.80	1.82	2.609 (3)	170
N1—H1A···Cl1^iii^	0.90	2.64	3.5028 (17)	162
N1—H1B···Cl1^iv^	0.90	2.60	3.3978 (17)	148

Symmetry codes: (ii) −*x*+3/2, −*y*+3/2, −*z*+2; (iii) −*x*+1, −*y*, −*z*+2; (iv) −*x*+1, *y*−1, −*z*+3/2.
